# The Effect of Antioxidant Supplementation on Fatigue during Exercise: Potential Role for NAD^+^(H)

**DOI:** 10.3390/nu2030319

**Published:** 2010-03-10

**Authors:** John Mach, Adrian W. Midgley, Steve Dank, Ross S. Grant, David J. Bentley

**Affiliations:** 1 School of Medical Science, University of New South Wales, Kensington, 2052, Australia; Email: John.mach@unsw.edu.au (J.M.); r.grant@unsw.edu.au(R.S.G.); 2 Department of Sport, Health and Exercise Science, University of Hull, Hull, HU6 7RX, UK; Email: A.W.Midgley@hull.ac.uk; 3 Department of Pharmacology, University of Sydney, Sydney, 2006, Australia; Email: steve.dank@gmail.com; 4 Australasian Research Institute, Sydney Adventist Hospital, Sydney, 2076 Australia;; 5 Health and Exercise Science, University of New South Wales, Kensington, 2052 Australia

**Keywords:** NAD^+^, NADH, exercise, oxidative stress, antioxidant

## Abstract

This study compared serum pyridine levels (NAD^+ ^/NADH) in trained (n = 6) and untrained (n = 7) subjects after continuous progressive exercise at 50%, 70% then 95% of physical work capacity until fatigue (TTF) after consuming a placebo or antioxidant (AO) cocktail (Lactaway^©^). An increase of 17% in TTF was observed in AO as compared to placebo (p = 0.032). This was accompanied by a significant increase in serum NAD^+^ levels (p = 0.037) in the AO supplemented group post exercise. The increases in NAD^+^ and improved endurance reflect lower oxidative stress-induced suppression of aerobic respiration.

## 1. Introduction

Fatigue can be defined as the reversible decline in skeletal muscle contractile performance due to intense muscle activity [1]. Fatigue is associated with many physiological factors including reduced neural input (central & peripheral) and disruptive metabolic changes in skeletal muscle such as lactic acidosis and the production of oxidative free radicals [1]. 

During high-intensity exercise the rate of ATP hydrolysis may exceed the rate of resynthesis resulting in reduced ATP levels and muscle fatigue. Studies examining human skeletal muscle indicate that before exhaustion, the electron transporter NADH increases [2,3], while muscle [4] and blood NAD^+^ levels decrease [5,6]. This indicates that the rate of NAD^+^ reduction (to form NADH) is outpacing its reoxidation during the ATP production process. An increase in NADH levels in the presence of decreased ATP (as occurs in fatigue) suggests the transfer of electrons from NADH may be compromised, thus impeding muscle performance resulting in fatigue.

The increase in oxidative stress (by the production of free radicals) could be one of the many factors affecting electron transfer from NADH. Exhaustive exercise increases the level of oxidative stress in the exercised tissue [7]. These radicals are very reactive resulting in inhibition of the tricarboxylic acid (TCA) and electron transport chain [8,9,10,11]. This includes NADH dehydrogenase (complex I) which plays the key role in oxidising NADH to NAD^+^[12,13]. Thus, elevated oxidative stress during high intensity exercise may suppress the respiratory chain, reducing the efficiency of NADH:NAD^+^ cycling and energy (ATP) production. ATP synthesis may therefore be improved during exercise by reducing oxidative stress through the use of antioxidant supplementation. 

Pycnogenol (PYC) is a mixture of procyanidins extracted from the bark of the pine, *pinus maritime*, which has an antioxidant capability. Studies have shown that pycnogenol contains many constituents with free radical scavenging properties, including polyphenols and flavonoids [14]. Additionally, pycnogenol inhibits free radical producing enzymes and stimulates the expression of antioxidant enzymes [14,15].

One study performed in this laboratory examining the effect of supplementation of antioxidants including pycnogenol found a 21% increase in time to fatigue during exhaustive exercise in humans [16]. The mechanism of this effect is unknown, but could be due to the antioxidant protection provided to muscular components and antioxidant action that allows the mitochondria to function normally, facilitating energy production from NADH. To our knowledge, no study currently exists on the effect of antioxidant administration on blood NAD^+ ^and NADH levels after exercise. 

The effectiveness of antioxidant supplementation on physical performance may be influenced by fitness level. Skeletal muscle possesses a number of mitochondrial or sarcoplasmic based enzymes such as CuZn-superoxide dismutase (CuZn-SOD; SOD1), catalase, and glutathione peroxidise that serve to resist oxidative stress [17]. Researchers have found that exercise stimulates the expression of antioxidant enzymes [18]. Trained individuals may therefore have a greater antioxidant capacity compared to untrained. Hence, the supplementation of an antioxidant may show greater benefits in untrained subjects compared to trained subjects. To our knowledge no study has previously investigated this phenomenon, necessitating this current research.

The purpose of the present study was to determine the effects of an antioxidant supplement (AO) containing pycnogenol on high intensity exercise performance in trained and untrained subjects. We also examined the potential mechanism of the effect by measuring NAD^+^ and NADH before and after exercise. We hypothesised that acute supplementation with the AO prior to a task of short, intensive exercise would enhance endurance performance and increase the efficiency of NADH transfer. Furthermore, the effect would be greater in untrained subjects.

## 2. Methodology

### 2.1. Subjects

Trained (n = 6) and untrained (n = 7) males with mean ± SD ages of 30 ± 6 and 29 ± 7 years and body masses of 79.5 ± 10.8 and 74.9 ± 5.2 kg, respectively, volunteered for the study. The trained subjects were cyclists or triathletes who participated on average in more than 3 sessions of cycle training per week, whilst the untrained subjects were sampled from the general population, were asymptomatic of any pathology but did not participate in any form of exercise training. All subjects provided informed consent before participating in the study. This study was approved by the University of New South Wales Human Research Ethics Committee. Subjects completed a medical questionnaire identifying contraindications to exercise. No subjects were taking medication or dietary supplements at the time of the study. The subjects were advised to maintain their normal dietary intake for the period in which the experiments were run and to avoid intake of antioxidant rich food. Subjects were healthy and were required to refrain from exercise 48 hours prior to the experiment and to fast two hours before. 

### 2.2. Experimental Protocol

Subjects visited the laboratory for testing on four occasions: (1) incremental exercise test; (2) time-to-fatigue familiarisation trial with no supplement (; time-to-fatigue trial with an AO-containing supplement (SUPPL); and (3) time-to-fatigue trial with a placebo (PLAC). The incremental exercise test enabled determination of peak power output (PPO) to standardize the exercise workload in the following trials. The familiarisation trial was included in the study to reduce any learning effects. The final two trials were conducted in a double-blinded fashion.

All exercise tests were conducted in a controlled environment (temperature ~20 °C, relative humidity ~50%) on a stationary braked cycle ergometer (Danpri, Melbourne, Australia) equipped with a power meter enabling measurements of power output (Schoberer Rad Messtechnik, Science, Julich, Germany). This device was calibrated as previously described [19] and adjusted to suit each subject’s physical requirements and normal cycling set-up (for the trained subjects). A telemetric heart rate monitor was used to record heart rate throughout exercise (Polar T31, Polar, Kempele, Finland). Subjects were allowed several days rest between experiments to avoid training adaptations affecting the study.

### 2.3. Incremental Exercise Test

Subjects performed a progressive exercise test to exhaustion on the cycle ergometer. Peak power output was determined by increasing the work rate by 30 W every 3 min, whilst maintaining pedal cadence at approximately 70 rpm. The initial work rate was determined by calculations based on the weight and fitness level of the subject (2 W·kg^-1^ for trained, 1 W·kg^-1^ for untrained) [20]. Exhaustion was defined as the subject’s inability to maintain the required cadence after three attempts of verbal support. Peak power output was quantified as the highest average output within a 3 min interval prior to fatigue [20].

### 2.4. Familiarisation

Subjects performed the cycle exercise in a step-wise manner beginning at 50% PPO for 4 min, followed by 70% PPO for 8 min, and then cycling to exhaustion at 95% PPO (time to fatigue) until they were unable to maintain the required cadence [21]. Throughout each trial subjects were not aware of the elapsed time or any physiological measures.

Venous blood samples were drawn via venipuncture before and immediately after exercise into ethylenediaminetetraacetic acid (EDTA) tubes. Fingertip capillary blood samples were also extracted to determine blood lactate concentration at the end of each exercise stage (50% PPO, 70% PPO and immediately upon fatigue at 95% PPO).

### 2.5. Placebo and Supplement Trials

Subjects consumed 150 ml of either the PLAC or SUPPL (Lactaway^©^, Away Australia Pty Ltd, Sydney, Australia (in a randomised order) with the same pre-test meal three hours before starting each experimental trial. The SUPPL and PLAC were the same in taste and appeared similar with observation. The SUPPL contained the exact energy content (315 KJ) and constituents (Pineapple Pulp, Molasses, Sodium Chloride, Flavour, Steviol Glycosides, Sodium Benzoate, Potassium Sorbate) of PLAC (315 KJ) with the exception of 0.36 mg of pycnogenol. The pycnogenol dosage was selected as pilot testing has shown it to optimise gastrointestinal discomfort and increasing positive physiological effects.

### 2.6. Blood Collection and Analysis

Blood lactate was measured using a portable device (Lactate Pro, Arkray. Inc, Tokyo, Japan). The blood samples were analysed immediately after the sample was taken. Whole blood samples were collected *via* venipuncture in EDTA and finger prick in heparin tubes, and centrifuged at 3,000 rpm to allow plasma to be isolated. Blood serum was extracted and stored at 4 °C immediately after centrifugation for NAD^+^(H) cycling assay and tested within 2 hours.

NAD^+^, NADH and total NAD^+^(H) levels were determined using a modified version of the NAD^+^(H) thiazolyl blue microcycling assay described by Bernofsky and Swan [22]. The NADH levels were determined using the total NAD^+^(H) reaction mixture without alcohol dehydrogenase and ethanol (table 4) eliminating the influence of the conversion of NAD^+^ to NADH on the sample NADH levels. This was included with a NADH standard to standardise the results and accurately determine NADH levels. 

Lipid peroxidation was determined by the quantification of the level of Thiobarbituric Acid Reactive Substances (TBARS) in the human plasma using a standardised commercial assay kit (Caymen Chemical Co. Ann Arbor, MI USA).

### 2.7. Statistical Analysis

All statistical analyses were performed using PASW statistics 17 (SPSS Inc., Chicago, IL), unless otherwise stated. Quantile-quantile plots indicated that the normality assumption for NAD+, NADH, NADH+(H), TBARS, power, heart rate and VO_2_ was plausible. However, time to fatigue and blood lactate concentration exhibited right skewed distributions, which were largely corrected using Box-Cox transformations. A Box-Cox transformation analysis was used to obtain the optimal value of lambda for each transformation (MINTAB version 15.1, Minitab Inc., State College, PA). The effects of condition (PLAC and SUPPL) and group (untrained and trained) on the change in each dependent variable were analysed using linear mixed models. Time also was modelled for NAD+, NADH and NADH+(H) using the change scores, and work rate (pre-exercise, and 50%, 70% and 95% PPO) also was modelled for blood lactate concentration. Condition, group and time were modelled as fixed effects and subject as a random effect. Various covariance structures were assumed and the one that minimised the Hurvich and Tsai’s criterion (AICC) value was chosen for the final fitted model for each dependent variable. Two-tailed statistical significance was accepted as p < 0.05.

## 3. Results

The median (interquartile range) of the untransformed data for the PLAC and SUPPL condition were respectively, 308 (269) and 369 (228) s for the untrained group and 400 (197) and 491 (97) s for the trained group. A significant main effect for condition for time to fatigue at 95% PPO was observed (F = 6.1, p = 0.032), where the time to fatigue was 17% higher in SUPPL compared to PLAC. There was no main effect for group (F = 0.6, p = 0.47) and no significant condition x group interaction effect (F = 0.03, p = 0.88).

A significant main effect for work rate was observed for blood lactate concentration (F = 216.2, p < 0.001). As would be expected, blood lactate continually increased as subjects went from rest to 95% PPO. However, no statistical significances were observed for the main effects of condition (F = 0.3, p = 0.61) and group (F = 1.1, p = 0.31), or the condition x work rate interaction (F = 0.3, p = 0.79), the condition x group interaction (F = 0.6, p = 0.44), the work rate x group interaction (F = 2.0, p = 0.16), or the condition x work rate x group interaction (F = 1.4, p = 0.26).

There was a significant main effect for condition for ΔNAD^+^ (F = 6.6, p = 0.037) where the NAD^+ ^increased in SUPPL but exhibited a decrease in PLAC (Figure 2). No statistical significance was observed for the main effect for group (F = 0.002, p = 0.96), or the condition x group interaction (F = 0.4, p = 0.52). The main effect for condition (F = 2.4, p = 0.16) and group (F = 3.1, p = 0.12), and the condition x group interaction (F = 0.07, p = 0.80) for ΔNADH were not statistically significant (Figure 3). Similarly, the main effects for condition (F = 5.4, p = 0.054) and group (F = 0.6, p = 0.47), and the group x condition interaction (F = 1.0, p = 0.36) for ΔNADH^+^(H) were not statistically significant.

**Figure 1:**
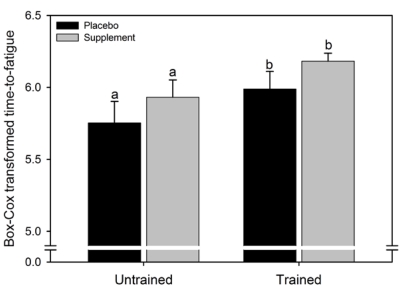
Mean (SEM) time to fatigue at 95% peak power output for the two experimental conditions. The data were transformed to correct for positive skewness. Like letters above error bars represent significant differences between conditions (p < 0.05).

**Figure 2:**
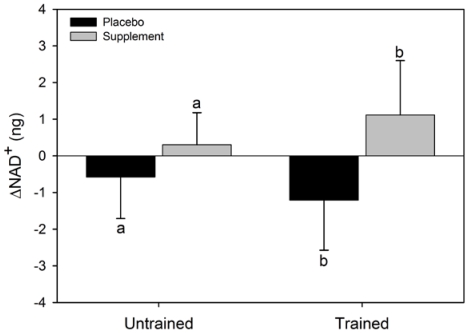
Mean (SEM) change in NAD^+^ for untrained and trained subjects during the two experimental conditions. Like letters above and below error bars represent significant differences between conditions (p < 0.05).

**Figure 3:**
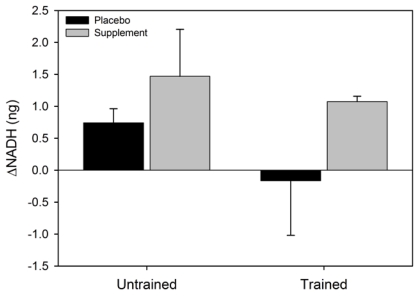
Mean (SEM) change in NADH for untrained and trained subjects during the two experimental conditions.

**Figure 4:**
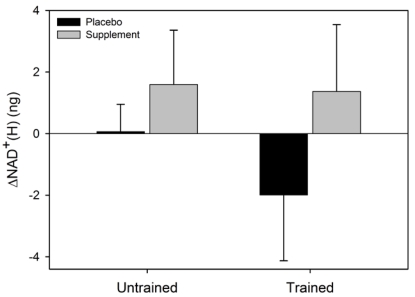
Mean (SEM) change in total NAD^+^(H) for untrained and trained subjects during the two experimental conditions.

No statistical significance was observed for the main effects of condition (F = 3.4, p = 0.1) and group (F = 1.9, p = 0.2) for VO_2_; however, there was a significant condition x group effect (F = 11.8, p = 0.01). The VO_2_ was significantly higher in SUPPL than in PLAC for the trained group (p = 0.004), but no significant difference was observed for the untrained group (p = 0.33).

No significant main and interaction effects were observed for TBARS, power and heart rate (p ≥ 0.05 for all F ratios) (Table 1).

**Table 1:** Mean (SD) TBARS before and after the two experimental conditions, and mean (SD) power, heart rate and VO_2_ during the two experimental conditions.

		Untrained		Trained	
		Placebo	Supplement	Placebo	Supplement
TBARS#	Pre	5.9 (2.5)	5.5 (0.6)	4.9 (2.0)	7.5 (2.5)
	Post	7.2 (4.1)	5.8 (1.2)	5.9 (2.0)	8.6 (1.0)
Power (W)		296 (29)	295 (29)	348 (17)	348 (16)
Heart rate (bpm)		183 (11)	182 (16)	178 (9)	179 (8)
VO_2_ (mL/min)		4210 (493)	4140 (593)	4658 (271)*	4850 (352)*

# Malondialdehyde equivalents. * p = 0.004.

## 4. Discussion

The main aim of the present study was to investigate the effects of ingesting an antioxidant complex, Lactaway^©^ containing pycnogenol, on exercise capacity and redox state. Pycnogenol has exceptional antioxidant capabilities [14] and has the capacity to provide intracellular and intramitochondrial antioxidant protection [15] and thus the potential to delay physical fatigue.

The present study demonstrated that ingestion of the AO supplement containing pycnogenol prior to short intensive exercise increases time to fatigue at 95% PPO by 17% on average. This is consistent with Bentley *et al.* [19] who found that supplementation with Lactaway^©^ improved time to fatigue during exercise at 95% VO_2_max by ~15% compared to a placebo conditions. The key novel finding in the present study is that the ingestion of the AO supplement was associated with an increased plasma NAD^+^ level during exercise, providing support for the premise that pharmacological modulation of redox cycling during exercise may result in improvements in physical performance.

NAD^+^ is a major cofactor in the transfer of electrons for ATP resynthesis. Maintenance of NAD^+^ levels is therefore required for sustained muscle activity. Previous studies have shown that NAD^+^ levels rise in the blood after moderate exercise and decrease in blood and muscle tissue after intensive exercise [4,5,6]. Consistent with these findings, we also observed a decrease in serum NAD^+ ^levels after intensive exercise in the experimental conditions where no AO was ingested.

During cellular respiration NAD^+^ is reduced to NADH by the enzymes of glycolysis and the tricarboxylic acid (TCA) cycle before being re-oxidised to NAD^+^ by cytochrome complexes in the mitochondria. Sahlin and colleagues, using muscle biopsies, found that NADH levels increased during intense exercise [2,3], consistent with a reduction in electron transfer from NADH down the respiratory chain. This is most likely due to accumulated ROS damage to mitochondrial complex 1 and possibly other complexes [22]. We also observed an increase in serum NADH following intense exercise in untrained subjects consistent with a trend toward increasing lipid peroxidation (Table 1). In contrast, we observed a decrease in serum NADH levels in athletes following intense exercise. This discrepancy is consistent with a previous report showing that athletes have greater cellular antioxidant capacity than untrained individuals [18]. The observed increase in serum NADH in untrained subjects and decreased NADH in athletes in the present study may therefore be due to relative differences in the impact of oxidative stress on complex 1 activity. If the exercise-induced increase in oxygen radicals exceeds the cell’s antioxidant capacity then considerable reductions in complex 1 activity will occur [23]. Decreased complex 1 activity results in reduced NADH oxidation and increased NADH accumulation, as seen in our untrained subjects. This results in reduced ATP production and resultant fatigue. Skeletal muscle ATP levels may therefore be effectively maintained by reducing oxidative stress induced mitochondrial suppression, thereby promoting sustained muscle activity. 

Following the ingestion of the AO, we observed a significant increase in NAD^+^ in both trained and untrained subjects. This observed increase may have resulted from reduced NAD^+^ catabolism. It is well established that oxidative stress increases the activity of the NAD^+^ consuming base excision repair enzyme poly(ADP-ribose) polymerase (PARP) [24]. A pycnogenol -mediated decrease in cellular ROS will reduce PARP activation and therefore NAD^+ ^catabolism. More NAD^+^ will therefore be available to act as substrate for reduction to NADH facilitating ongoing energy (ATP) synthesis in the mitochondria, providing a physiological basis for the observed improvement in TTF in the present study.

In conclusion, the present study has shown that blood NAD^+^ levels decrease following intense exercise. NADH levels increased in untrained subject but decreased in trained athletes, most likely because differences in antioxidant capacity and resultant ROS affected NAD^+^/NADH cycling in the mitochondria. For the first time we have also shown that the observed increase in time to fatigue for subjects ingesting an AO supplement was accompanied by an increase in serum NAD^+^ levels. The associated increases in both oxidised and reduced NAD^+^ and time to fatigue following AO supplementation is consistent with the hypothesis that AO supplementation reduces the oxidative stress induced suppression of aerobic respiration within the mitochondria, thereby increasing the efficiency of electron transfer and ATP production.

## Supplementary Files

Supplementary File 1: Correction (PDF, 169 KB)
