# Correction: Mach, J., *et al*. The Effect of Antioxidant Supplementation on Fatigue during Exercise: Potential Role for NAD^+^(H). *Nutrients* 2010, *2*, 319-329

**DOI:** 10.3390/nu2040481

**Published:** 2010-04-13

**Authors:** John Mach, Adrian W. Midgley, Steve Dank, Ross S. Grant, David J. Bentley

**Affiliations:** 1 School of Medical Science, University of New South Wales, Kensington, 2052, Australia; Email: John.mach@unsw.edu.au; 2 Department of Sport, Health and Exercise Science, University of Hull, Hull, HU6 7RX, UK; Email: A.W.Midgley@hull.ac.uk; 3 Department of Pharmacology, University of Sydney, Sydney, 2006, Australia; Email: steve.dank@gmail.com; 4 Australasian Research Institute, Sydney Adventist Hospital, Sydney, 2076, Australia; Email: r.grant@unsw.edu.au; 5 Health and Exercise Science, University of New South Wales, Kensington, 2052, Australia

We have found an error in our manuscript published in *Nutrients* [1]. On page 322 in the methods (section 2.5) it states ‘0.36 mg’ of pycnogenol is contained in one dose of the lactaway (antioxidant) supplement. This should in fact read ‘36 mg’ of pycnogenol. This is purely a typographical error and does not impact on the results or conclusions drawn from this work. However we do apologise for any inconvenience caused to the readers.

## References

[1] Mach J., Midgley A.W., Dank S., Grant R.S., Bentley D.J. (2010). The Effect of Antioxidant Supplementation on Fatigue during Exercise: Potential Role for NAD+(H). Nutrients.

