# Supplementary material for: The Effect of Antioxidant Supplementation on Fatigue during Exercise: Potential Role for NAD+(H)
Source: Nutrients. 2010 Mar 10;2(3):319–29. doi: 10.3390/nu2030319 (PMC3257644; doi:10.3390/nu2030319)
Supplement: Supplementary File 1: — Correction (PDF, 169 KB) [file nutrients-02-00319-s001.pdf]

Nutrients **2010**, *2*, 481; doi:10.3390/nu2040481

OPEN ACCESS

nutrients

ISSN 2072-6643

www.mdpi.com/journal/nutrients

Correction

Correction: Mach, J., *et al.* The Effect of Antioxidant Supplementation on Fatigue during Exercise: Potential Role for NAD$^+$(H). *Nutrients* 2010, *2*, 319-329

John Mach [1], Adrian W. Midgley [2], Steve Dank [3], Ross S. Grant [1,4] and David J. Bentley [5,*]

1 School of Medical Science, University of New South Wales, Kensington, 2052, Australia; E-Mail: John.mach@unsw.edu.au

2 Department of Sport, Health and Exercise Science, University of Hull, Hull, HU6 7RX, UK; E-Mail: A.W.Midgley@hull.ac.uk

3 Department of Pharmacology, University of Sydney, Sydney, 2006, Australia; E-Mail: steve.dank@gmail.com

4 Australasian Research Institute, Sydney Adventist Hospital, Sydney, 2076, Australia; E-Mail: r.grant@unsw.edu.au

5 Health and Exercise Science, University of New South Wales, Kensington, 2052, Australia

* Author to whom correspondence should be addressed: E-Mail: david.bentley@adelaide.edu.au.

Received: 13 April 2010 / Published: 13 April 2010

We have found an error in our manuscript published in *Nutrients* [1]. On page 322 in the methods (section 2.5) it states '0.36 mg' of pycnogenol is contained in one dose of the lactaway (antioxidant) supplement. This should in fact read '36 mg' of pycnogenol. This is purely a typographical error and does not impact on the results or conclusions drawn from this work. However we do apologise for any inconvenience caused to the readers.

References

1. Mach, J.; Midgley, A.W.; Dank, S.; Grant, R.S.; Bentley, D.J. The Effect of Antioxidant Supplementation on Fatigue during Exercise: Potential Role for NAD+(H). *Nutrients* **2010**, *2*, 319-329.
